# Topological Phase and Strong Correlation in Rare-Earth Hexaborides XB_6_ (X = La, Ce, Pr, Nd, Pm, Sm, Eu)

**DOI:** 10.3390/ma13194381

**Published:** 2020-10-01

**Authors:** Sheng-Hsiung Hung, Horng-Tay Jeng

**Affiliations:** 1 Department of Physics, National Tsing Hua University, Hsinchu 30013, Taiwan; feynman0121@gmail.com; 2 Physics Division, National Center for Theoretical Sciences, Hsinchu 30013, Taiwan; 3 Institute of Physics, Academia Sinica, Taipei 11529, Taiwan

**Keywords:** topological phase, strong correlation, Hexaboride, first-principles calculations, electronic structures

## Abstract

The rare-earth hexaboride SmB_6_, known as the topological Kondo insulator, has attracted tremendous attention in recent years. It was revealed that the topological phase of SmB_6_ is insensitive to the value of on-site Coulomb interactions (Hubbard U), indicating that the topological phase in SmB_6_ is robust against strong correlations. On the contrary, the isostructural YbB_6_ displays a sensitivity to the Hubbard U value. As U increases, YbB_6_ transforms from topological Kondo insulator to trivial insulator, showing the weak robustness of the topological phase of YbB_6_ against U. Consequently, the dependence of the topological phase on Hubbard U is a crucial issue in the rare-earth hexaboride family. In this work, we investigate the structural and electronic properties of rare-earth hexaboride compounds through first-principles calculations based on density functional theory. By taking the strong correlations into consideration using a wide range of on-site U values, we study the evolution of the topological phases in rare-earth hexaboride (XB_6_, X = La, Ce, Pr, Nd, Pm, Sm, Eu). Unlike YbB_6_, the topological trends in all the examples of XB_6_ studied in this work are insensitive to the U values. We conclude that in addition to the well-known SmB_6_, PmB_6_, NdB_6_ and EuB_6_ are also topologically nontrivial compounds, whereas LaB_6_, CeB_6_ and PrB_6_ are topologically trivial metal.

## 1. Introduction

The discovery of the topological phase in condensed matter paved the way to classify electronic states [1,2]. Topological insulators have been attracting world-wide extensive attention in recent research [3,4,5,6]. Three dimensional materials with time reversal symmetry and inversion symmetry may harbor a topologically nontrivial phase if a band gap and band inversion emerge owing to spin–orbit interaction (SOI) [7].

**Figure 1:**
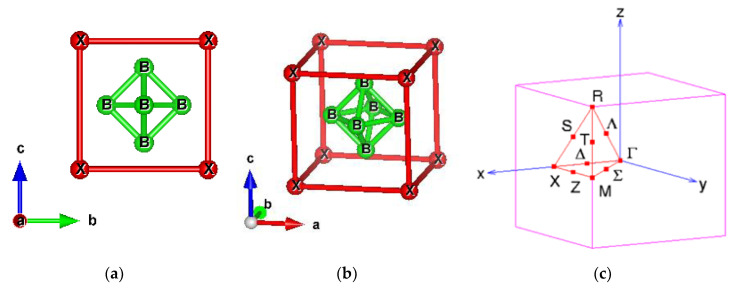
CsCl-type cubic crystal structure and Brillouin Zone of rare-earth hexaboride XB_6_. (**a**) Side view. (**b**) Oblique view. (**c**) Brillouin Zone and high symmetry k-points.

The rare earth hexaboride XB_6_ crystallizes in the CaB_6_-structure, as shown in Figure 1. Its lattice structure is similar to a body-centered cubic such as the CsCl-type lattice with Cs replaced by rare earth ions, and with Cl substituted by B_6_ octahedra. The variety of the physical properties observed in these compounds is intriguing. For example, the application of LaB_6_ has been paid attention due to its low work function, which is suitable for thermionic emission. LaB_6_ is metallic and becomes superconducting at T_C_ = 0.45 K [8]. CeB_6_ is considered as a Kondo system. CeB_6_ presents an antiferro-quadrupolar ordering in the paramagnetic phase between T_q_ = 3.3 K (quadrupolar ordering temperature) and T_N_ = 2.4 K (Neel’s Temperature) [9,10]. PrB_6_ has been confirmed that negative quadrupolar pair interactions exist in the paramagnetic phase (T_N_ = 6.9 K) [11]. NdB_6_ is a localized 4f system that orders ferro-magnetically at low temperatures [12]. SmB_6_ is a well-known topological Kondo insulator [1,13,14]. EuB_6_ orders ferro-magnetically below 15.1 K with a huge decrease of resistivity and a significant blue shift of the reflectivity plasma edge [15,16,17]. At 12.7 K, another phase transition takes place, which is observed as a broad peak in the specific heat or an anomaly in the resistivity [18]. GdB_6_ is a localized 4f system with a ferromagnetic order at low temperatures [19]. YbB_6_ is a topology Kondo insulator at low temperatures, and is a classical mixed valence narrow band gap semiconductor [1,20,21]. Structural studies are also presented in Ref. [22,23]. As reported in Ref. [1], the topological phase of YbB_6_ is sensitive to the Hubbard U value. As U increases, YbB_6_ transforms from topological Kondo insulator to trivial insulator, showing the weak robustness of the topological phase of YbB_6_ against U.

In this study, the lattice structures of rare-earth hexaboride (XB_6_, X = La, Ce, Pr, Nd, Pm, Sm, Eu) are fully optimized through first-principles calculations. We then perform self-consistent field electronic structure calculations with and without SOI. To reveal the topological phases, we analyze if SOI would open up a continuous energy gap at the Fermi level with band inversion around the energy gap. To examine the robustness of the topological phase upon the strong correlation in XB_6_, we trace the evolution of its electronic structure by tuning the on-site U of the f electrons. We demonstrate that besides SmB_6_, PmB_6_, NdB_6_ and EuB_6_ are also topologically nontrivial compounds, while the others are topologically trivial normal metals.

## 2. Computational Details

First-principles calculations were performed using the Vienna Ab initio Simulation Package (VASP) with the Perdew-Burke-Ernzerhof (PBE) exchange-correlation functional used in the generalized gradient approximation (GGA) as well as the GGA plus Hubbard U (GGA + U) schemes [24,25,26,27] based on density functional theory (DFT). The cut-off energy of 500 eV was adopted for the plane-wave basis. A Γ-centered 15 × 15 × 15 k-mesh was used in geometry optimization and self-consistent field calculations. The geometry optimization converged until all residual forces remained below 0.01 eV/Å. Table 1 compares the experimental lattice parameters of rare-earth hexaboride with our geometrically optimized ones. Good agreement between experimental and theoretical results can be found with deviations, in general, of less than 1%.

## 3. Results and Discussion

### 3.1. Topologically Trivial Normal Metal LaB_6_, CeB_6_, and PrB_6_

**Figure 2:**
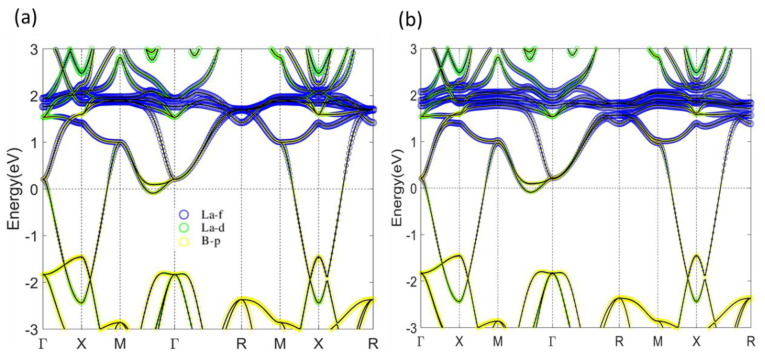
Atom-orbital decomposed band structure of LaB_6_ calculated using Perdew–Burke–Ernzerhof (PBE) functional without spin–orbit interaction (SOI) (**a**) and with SOI (**b**). The size of blue, green and yellow circles indicates components from La-f, La-d and B-p orbitals, respectively.

**Figure 3:**
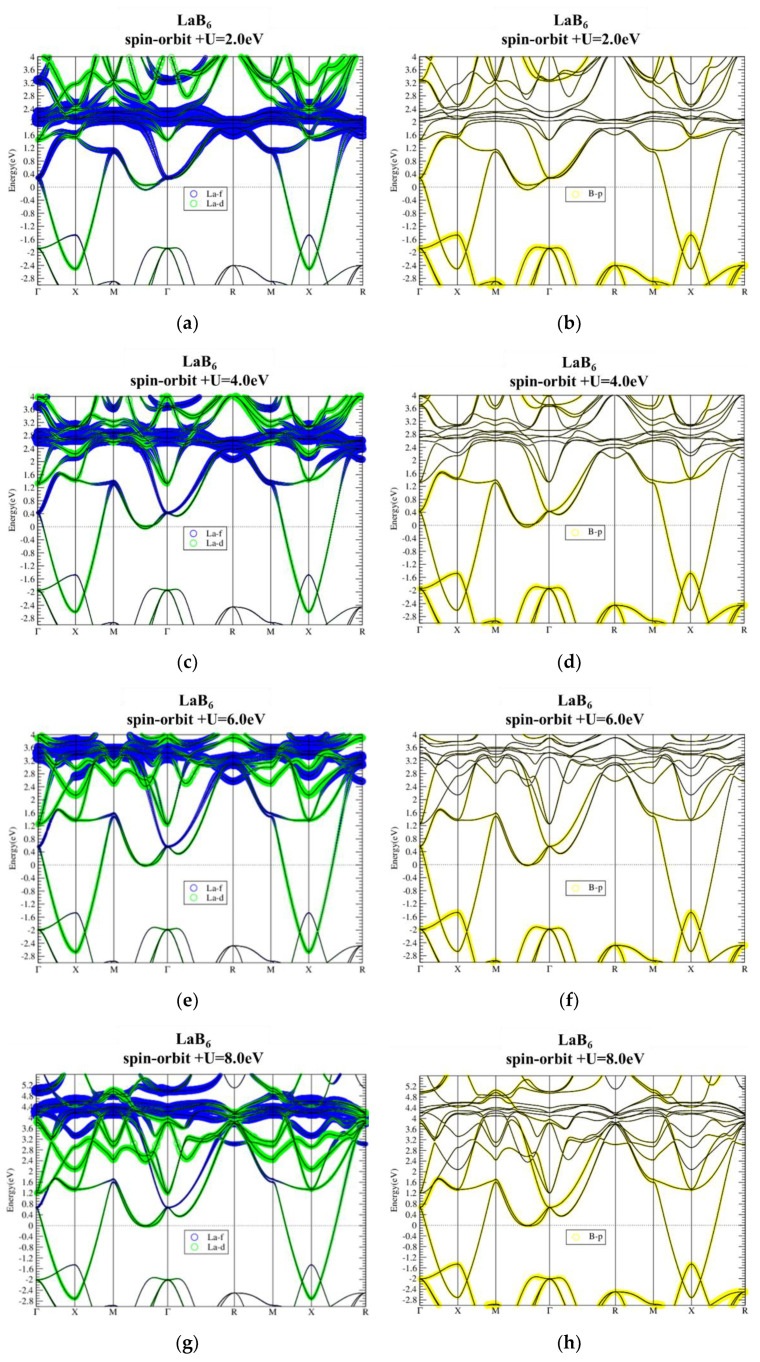
Atom-orbital decomposed band structures of LaB_6_ with on-site U =2 eV (**a**,**b**), 4 eV (**c**,**d**), 6 eV (**e**,**f**), and 8 eV (**g**,**h**).

Figure 2a,b show the PBE band structures of LaB_6_ without and with SOI, respectively. The atom-orbital decomposition demonstrates that the valence bands below −1.5 eV are mainly composed of B-p orbital, while the conduction La-f bands (blue curves) are located mainly from 0.5 eV to 2.5 eV above the Fermi level (E_f_). In between, there is a dispersive band composed of La-d orbital connecting the valence and conduction bands, resulting in an overall semimetal character. This in-gap La-d band also gives an electron pocket at E_f_ along ΓM. A comparison of band structures without SOI (a) and with SOI (b) shows that the SOI in LaB_6_ is weak and has no significant effect on band structure. Consequently, the semimetal character remains as SOI is included. Without any continuous gap, LaB_6_ is therefore a topologically trivial normal metal. On the other hand, the band structures remain more or less the same when the on-site Coulomb repulsion U is taken into account for the strong correlation in f orbitals, as can be seen in Figure 3. This constitutes preassembly, owing to the empty f states that have no effect near E_f_.

**Figure 4:**
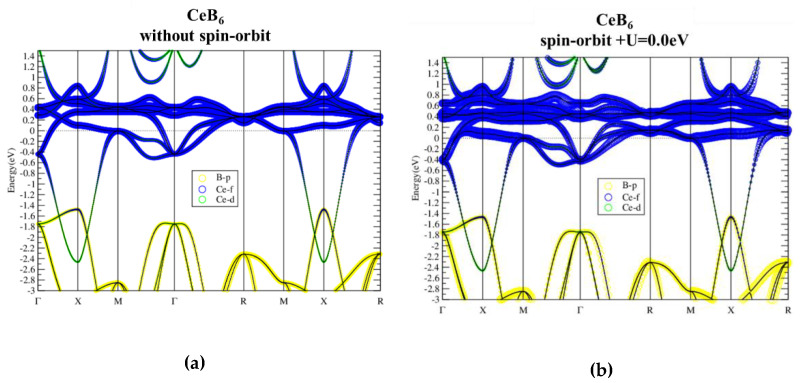
Band structure of CeB_6_ without (**a**) and with (**b**) spin–orbit interaction.

**Figure 5:**
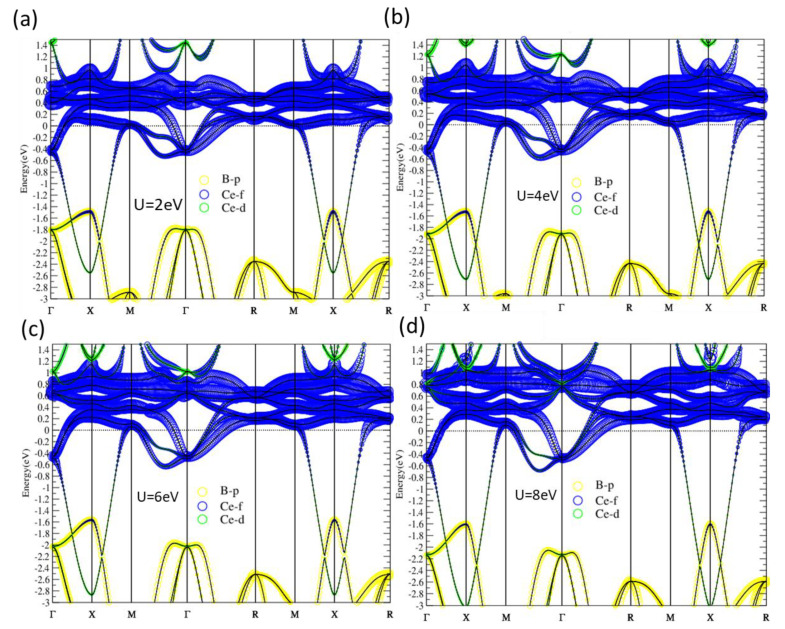
(**a**–**d**) CeB_6_ band structures given from PBE + SOI + U with U = 2, 4, 6, 8 eV, respectively. The sizes of blue, green and yellow circles indicate components from Ce-f, Ce-d and B-p orbitals, respectively.

With one more electron than La, the Fermi level of CeB_6_ is thus raised up to the bottom of Ce-f bands, as shown in Figure 4. The flat Ce-f conduction bands are located around E_f_ from 0.6 eV below to 1.2 eV above E_f_. As shown in Figure 5, for all the four cases with U = 2, 4, 6, 8 eV studied, there are no significant changes in band structures. Similar to LaB_6_, CeB_6_ is also insensitive to the on-site U values. Although the SOI is included in the calculations and the degeneracy at M is lifted by SOI, there is no continuous gap in all cases, leading to topologically trivial normal metal ground state for CeB_6_.

**Figure 6:**
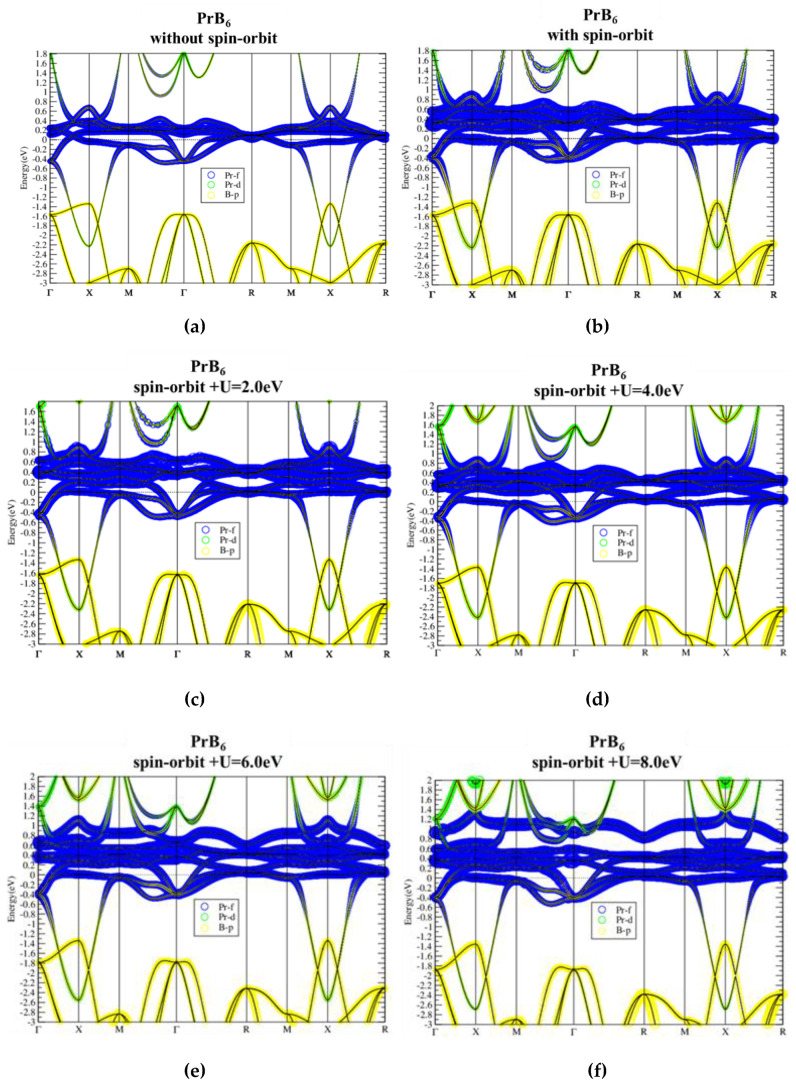
PrB_6_ band structure without (**a**) and with (**b**) spin–orbit interaction (noted in the figures), and with spin–orbit interaction plus on-site U = 2, 4, 6, 8 eV (**c**–**f**, respectively) as noted in the figures.

Elementary Pr has three electrons occupying the f-orbitals in the ground state. Therefore, in PrB_6_ the Pr-f conduction band is occupied by one more f electron than CeB_6_ through the rigid-band shift, as shown in Figure 6. The band dispersions remain similar with different on-site U values. However, because the Fermi level is raised to the middle of the Pr-f conduction band, on-site U affects the bandwidth more significantly than that in the previous two species. With U = 8.0 eV, the f bandwidth is enhanced by about 0.5 eV. On the other hand, gapless ground state remains in PrB_6_ even when the SOI is taken into consideration. Consequently, the same as LaB_6_ and CeB_6_, PrB_6_ is also a topologically trivial normal metal.

### 3.2. Topologically Nontrivial Kondo Insulator SmB_6_, PmB_6_, NdB_6_ and EuB_6_

**Figure 7:**
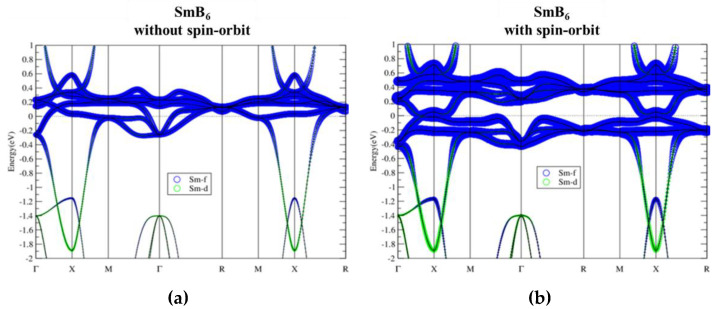
Band structures of SmB_6_ without (**a**) and with (**b**) spin-orbit interaction projected by f and d electrons of Sm.

**Figure 8:**
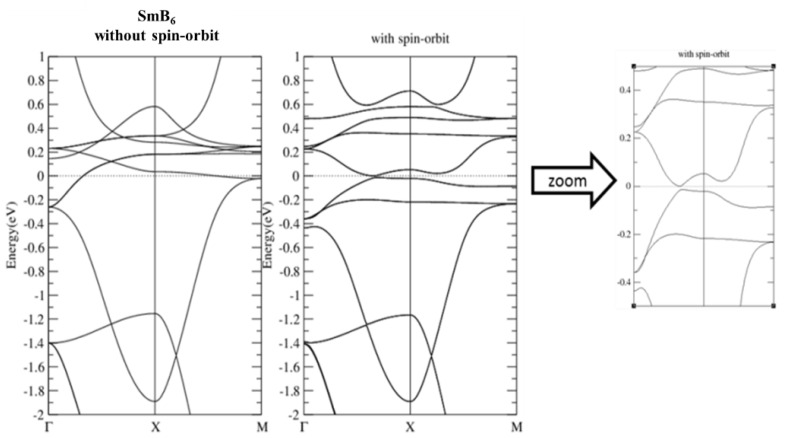
Band structure of SmB_6_ without and with spin-orbit coupling. The right panel is the zoom-in view of the middle panel around E_f_.

**Figure 9:**
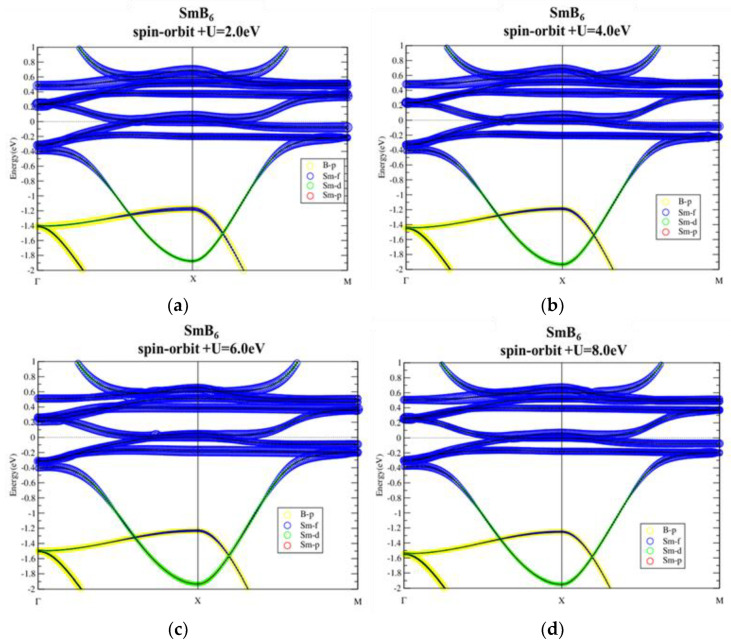
Band structures of SmB_6_ with spin–orbit interaction using on-site U = 2.0, 4.0, 6.0, and 8.0 eV (**a**–**d**, respectively) projected by f and d electrons of Sm.

Figure 7 shows our calculated band structures of the well-known topological Kondo insulator. The relatively flat La-f bands locate around Ef, with a much more dispersive La-d band crossing all these f bands. The spin–orbit interaction splits the f bands and opens up a continuous energy gap (see Figure 8) with band inversion between Sm-f/d characters flipping around the SOI-induced gap. These results agree well with those presented in previous works [1]. Band structures of SmB_6_ with SOI and on-site U ranging from 2 to 8 eV are shown in Figure 9. There are no significant changes in band structures due to all the different U values used. Similar to previous study, the SOI-induced band gap and the band inversion behavior remain, indicating the robust topological phase against strong correlations in SmB_6_.

**Figure 10:**
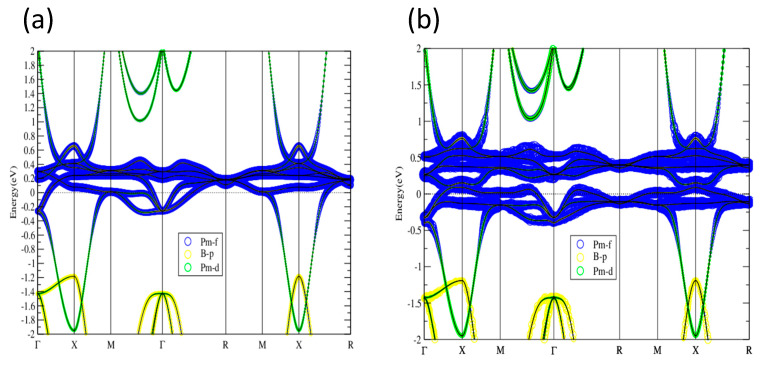

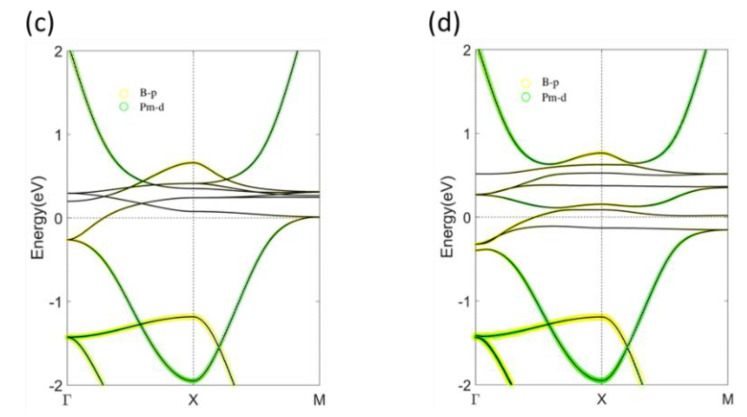
PBE band structure of PmB_6_ without SOI (**a**) and with SOI (**b**). The size of blue, green and yellow circles show contributions from Pm-f, Pm-d and B-p orbitals, respectively. (**c**) Zoom-in of (**a**). (**d**) Zoom-in of (**b**). (**c**,**d**) demonstrate SOI-induced band inversion and gap opening.

**Figure 11:**
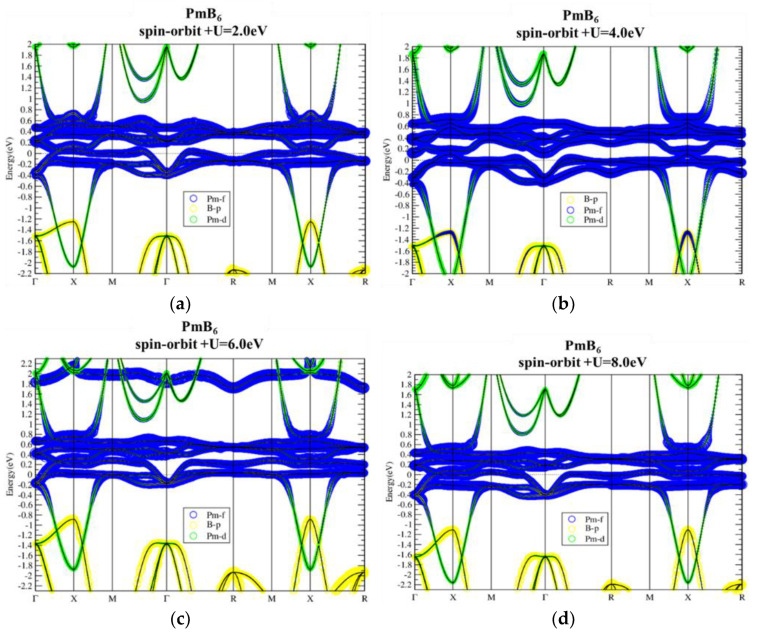
Band structures of PmB_6_ with SOI and U = 2, 4, 6, 8 eV (**a**–**d**, respectively). As U is tuned larger, the highest f band is lifted but the band property is not changed near the Fermi level.

In comparison with the well-known topological Kondo insulator SmB_6_, the overall band dispersion of PmB_6_ as shown in Figure 10 is similar to those of SmB_6_ (Figure 7, Figure 8 and Figure 9). Since PmB_6_ has one less valence electron than SmB_6_, the Fermi level of PmB_6_ is relatively lower than that of SmB_6_. With SOI taken into consideration, PmB_6_ opens up a continuous gap around Ef, as shown in Figure 10b,d. In addition, there is a band inversion around X point with B-p and Pm-d components exchanged near E_f_. Therefore, PmB_6_ can host topological nontrivial state, giving rise to the topological Kondo insulator similar to SmB_6_. The electronic structure of PmB_6_ around E_f_ is not sensitive to various U values, as shown in Figure 11. On-site U only affects the highest empty f-band without influencing the overall topological properties, indicating the topological phase is robust in PmB_6_ against strong correlations.

**Figure 12:**
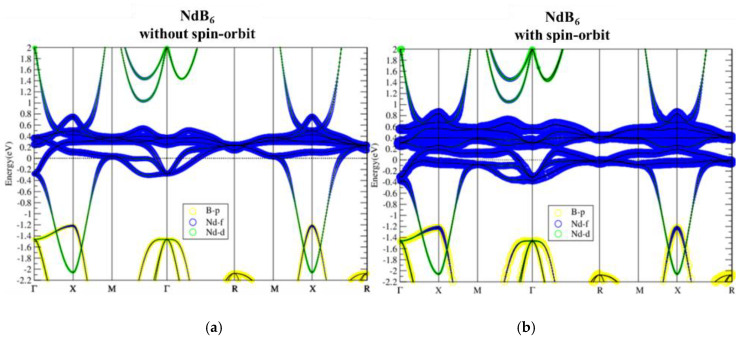
Band structures of NdB_6_ without (**a**) and with (**b**) spin-orbit interaction.

**Figure 13:**
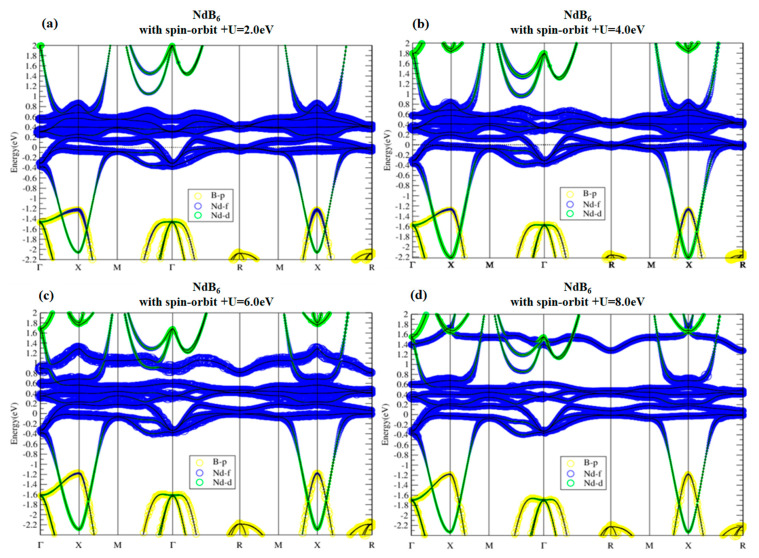
Band structures of NdB_6_ with SOI using U = 2, 4, 6, and 8 eV (**a**–**d**, respectively). As U is tuned larger, the highest f band is lifted but the band property is not changed near the Fermi level.

Band structures of NdB_6_ as shown in Figure 12 also demonstrate topologically nontrivial phase. The SOI not only opens up a continuous energy gap around E_f_ but also gives rise to band inversion around X point. Similar to PmB_6_, the electronic structure and topological behavior of NdB_6_ near the Fermi level are insensitive to on-site U value, as can be seen in Figure 13. Only the highest unoccupied f band is noticeably modified by U, which is irrelevant to its topology. Consequently, NdB_6_ is also a topological Kondo insulator.

**Figure 14:**
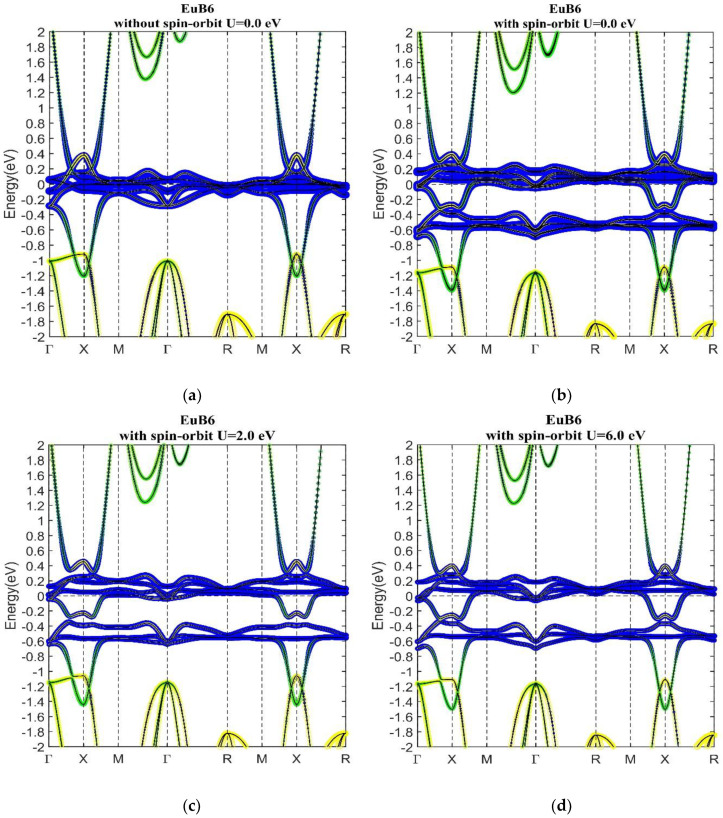
PBE band structure of EuB_6_ without SOI (**a**) and with SOI (**b**). SOI opens up an energy gap around E_f_ and induces band inversion around X point. PBE + U band structure of EuB_6_ with on-site U = 2.0 eV (**c**) and U = 6.0 eV (**d**). Similar to SmB_6_, the Hubbard U does not change the band structure noticeably.

Figure 14 shows PBE (U = 0 eV) band structures of EuB_6_ without and with SOI as well as PBE + U band structures with U = 2 eV and 6 eV. As can be seen in Figure 14a, the f bands are located at E_f_ with a localized flat band character. In the periodic table, Eu is the neighbor of Sm with one more electron. The additional electron raises the Fermi level of EuB_6_ near the half-filling metallic regime. When SOI is included, the f bands separate themselves into two groups with an SOI-induced continuous gap in between. Furthermore, band inversion emerges around the high symmetry point X. As a result, EuB_6_ exhibits nontrivial topological phase similar to SmB_6_. The band structure of EuB_6_ is not sensitive to U, as shown in Figure 14b–d, with U = 0–6 eV, leading EuB_6_ to robust topological Kondo insulator against strong correlations.

## 4. Conclusions

We have systematically analyzed the electronic structures of rare-earth hexaborides to investigate their topological properties and examine the robustness of the topological phase against strong correlations by varying the Coulomb repulsion U. SmB_6_ is a topological Kondo insulator due to the hybridization gap, and it will not experience topological phase transition by tuning the Coulomb interaction. YbB_6_, which has a hybridization gap, on the contrary, will experience a topology phase transition from a topological Kondo insulator to a topological insulator, and finally become a trivial insulator. Our results of SmB_6_ and YbB_6_ are in good agreement with previous results [1]. Our study also shows that PmB_6_, NdB_6_, EuB_6_ and SmB_6_ exhibit SOI-induced continuous gaps with band inversion, revealing nontrivial topological properties. On the other hand, the weaker SOI in relatively lighter. Lanthanides La, Ce and Pr fail to open up a continuous gap in LaB_6_, CeB_6_ and PrB_6_. Thus LaB_6_, CeB_6_ and PrB_6_ are topologically trivial normal metals with correlated conduction electrons.

## Figures and Tables

**Table 1:** Experimental (exp) and theoretical (the) lattice parameters. The rare-earth hexaboride crystalizes in a bcc-like structure with space group of $\mathrm{Pm}\bar{3}m$ (No. 221), in which metal ions are located at the Wyckoff position 1a(0,0,0) and octahedral B_6_ at the Wyckoff position 6f (1/2,1/2,z). The subscripts “exp” and “the” indicate experimental and theoretical results, respectively.

	a_exp_ (Å)	a_the_(Å)	Error of a	B(z)_exp_	B(z)_the_	B–B Bond Length_exp_(Å)	B–B Bond Length_the_(Å)
LaB_6_ [15]	4.1527	4.1553	0.06%	0.1993	0.1997	1.7660	1.7647
CeB_6_ [16]	4.14	4.1130	−0.65%	0.1992	0.1984	1.7611	1.7543
PrB_6_ [22]	4.13	4.1024	−0.67%	0.2	0.1984	1.7522	1.7498
NdB_6_ [16]	4.127	4.1007	−0.64%	0.1989	0.1987	1.7574	1.7473
PmB_6_ [23]	4.128	4.1131	−0.36%	0.2	0.1990	1.7514	1.7508
SmB_6_ [28]	4.1346	4.1087	−0.63%	0.2018	0.1993	1.7436	1.7474
EuB_6_ [29]	4.1849	4.1325	−1.25%	0.2027	0.1999	1.7595	1.7539
YbB_6_ [29]	4.1444	4.1325	−0.29%	0.207	0.2007	1.7173	1.7492
